# Comprehensive prognostic report of the Japanese Breast Cancer Society Registry in 2004

**DOI:** 10.1007/s12282-015-0644-5

**Published:** 2015-10-07

**Authors:** Takayuki Kinoshita, Naohito Fukui, Keisei Anan, Takayuki Iwamoto, Naoki Niikura, Masaaki Kawai, Naoki Hayashi, Kouichiro Tsugawa, Kenjiro Aogi, Takanori Ishida, Hideji Masuoka, Shinobu Masuda, Kotaro Iijima, Seigo Nakamura, Yutaka Tokuda

**Affiliations:** Division of Breast Surgery, National Cancer Center Hospital, Tokyo, Japan; The Japan Clinical Research Support Unit, Tokyo, Japan; Department of Surgery, Kitakyushu Municipal Medical Center, Kitakyushu, Japan; Department of Breast and Endocrine Surgery, Okayama University Hospital, Okayama, Japan; Department of Breast and Endocrine Surgery, Tokai University School of Medicine, 143 Shimokasuya, Isehara, Kanagawa 259-1193 Japan; Department of Breast Surgery, Miyagi Cancer Center, Natori, Japan; Department of Breast Surgery, St. Luke’s International Hospital, Tokyo, Japan; Division of Breast and Endocrine Surgery, Department of Surgery, St. Marianna University School of Medicine, Kawasaki, Japan; Department of Breast Surgery, Shikoku Cancer Center, Matsuyama, Japan; Department of Surgical Oncology, Graduate School of Medicine, Tohoku University, Sendai, Japan; Sapporo-Kotoni Breast Clinic, Sapporo, Japan; Department of Pathology, Nihon University School of Medicine, Tokyo, Japan; Department of Breast Oncology, Cancer Institute Hospital, Tokyo, Japan; Division of Breast Surgical Oncology, Department of Surgery, Showa University, Tokyo, Japan

**Keywords:** Breast cancer, Prognosis, Report, Japan, Registry, 2004, The Japanese Breast Cancer Society

## Preface

In 1975, the Breast Cancer Study Group (the predecessor of the Japanese Breast Cancer Society) initiated the Breast Cancer Registry and had registered 188,265 breast cancer patients during the 29 years from 1975 to 2003. In 2004, a new registration system was implemented, which had registered a total of 207,468 patients up to 2009.

When the Personal Information Protection Law came into effect in 2004, the previous paper-based registration system was converted to the web-based system, which includes anonymized efficacy data. At the same time, the data center was moved from the National Cancer Center to the Japan Clinical Research Support Unit (J-CRSU), a non-profit organization, and the Public Health Research Foundation.

Herein, we are pleased to report, for the first time under the new system, results on five-year prognosis of patients that were registered in 2004 (Figs. 1, 2, 3, 4, 5, 6, 7, 8, 9; Supplementary Tables 1–9). We are deeply grateful to the medical and administrative staff as well as the patients who co-operatively participated in this study.

**Fig. 1:**
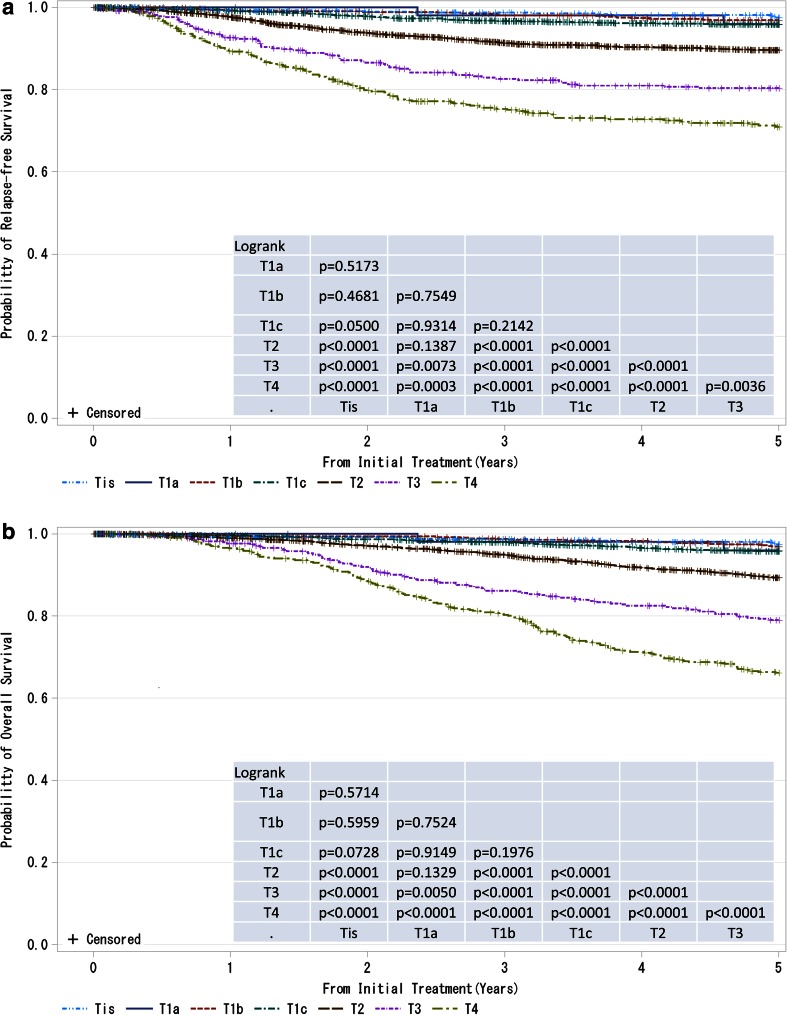
**a**, **b** Kaplan–Meier curves for relapse-free and overall survival of all cases by tumor classification (cT-category). *P* values were calculated using the log-rank test. Tis: Non-invasive ductal carcinoma, lobular carcinoma in situ, or Paget disease; T1a: ≤0.5 cm; T1b: 0.5 < tumor ≤ 1.0 cm; T1c: 1.0 < tumor ≤ 2.0 cm, T2: 2.0 < tumor ≤ 5.0 cm; T3: > 5.0 cm; T4: tumor of any size with direct extension to the chest wall and/or skin (ulceration or skin nodules) or inflammatory carcinoma

**Fig. 2:**
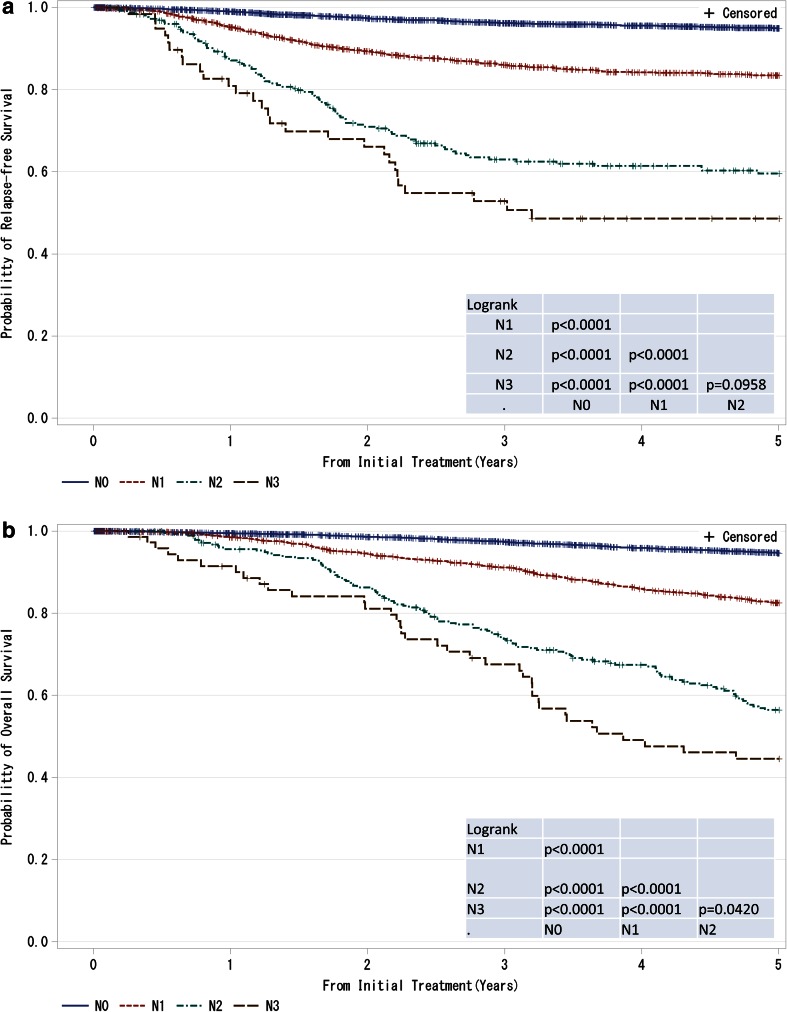
**a**, **b** Kaplan–Meier curves for relapse-free and overall survival of all cases by regional lymph nodes status (cN-category) N0: No regional lymph node metastases; N1: Metastases in movable ipsilateral level I, II axillary lymph node(s); N2: Metastases in ipsilateral level I, II axillary lymph nodes that are clinically fixed or matted OR Metastases in clinically detected ipsilateral internal mammary nodes in the *absence* of clinically evident axillary lymph node metastases; N3: Metastases in ipsilateral infraclavicular (level III axillary) lymph node(s) with or without level I, II axillary lymph node involvement OR Metastases in clinically detected ipsilateral internal mammary lymph node(s) with clinically evident level I, II axillary lymph node metastases OR Metastases in ipsilateral supraclavicular lymph node(s) with or without axillary or internal mammary lymph node involvement. *P* values were calculated using the log-rank test

**Fig. 3:**
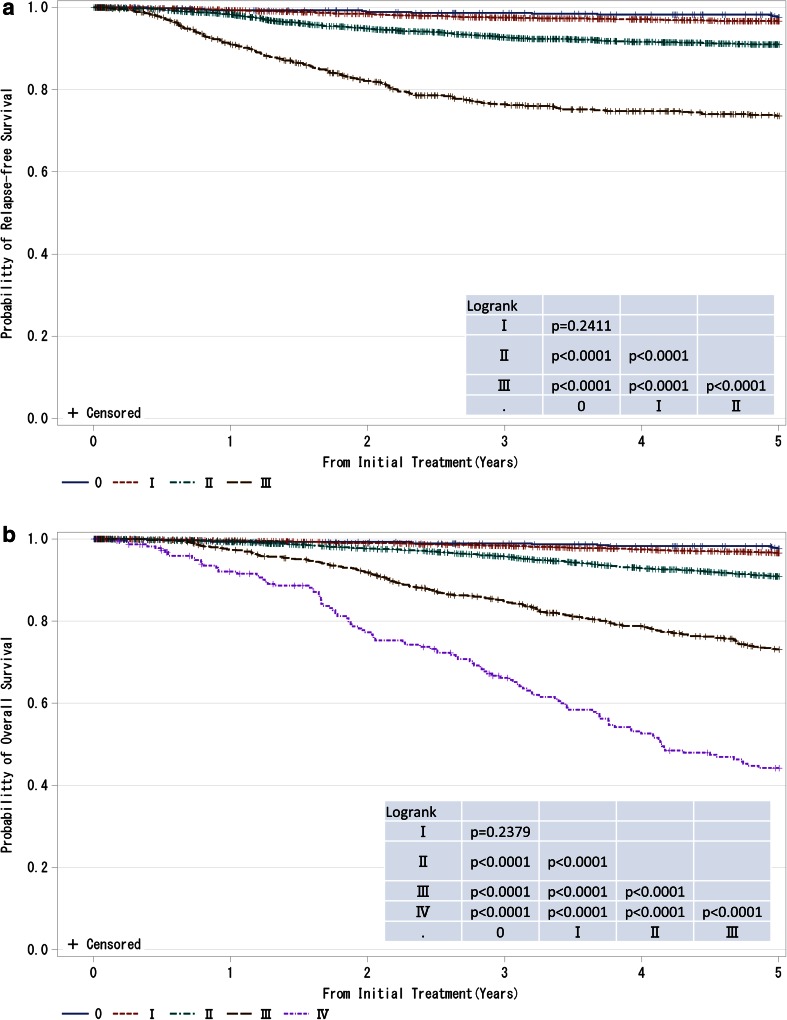
**a**, **b** Kaplan–Meier curves for relapse-free and overall survival of all cases by clinical stage (UICC). *P* values were calculated using the log-rank test

**Fig. 4:**
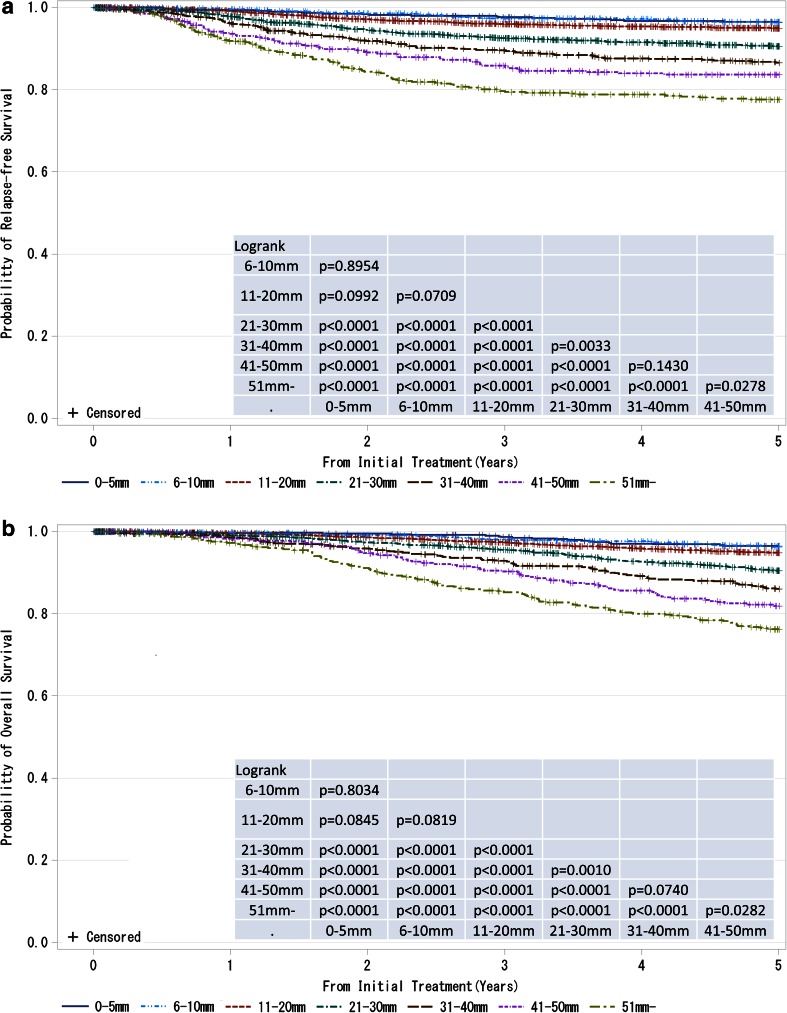
**a**, **b** Kaplan–Meier curves for Relapse-free and overall survival of cases without neoadjuvant therapy by pathological tumor size (pT size). Tumor size is a marker of invasiveness. *P* values were calculated using the log-rank test

**Fig. 5:**
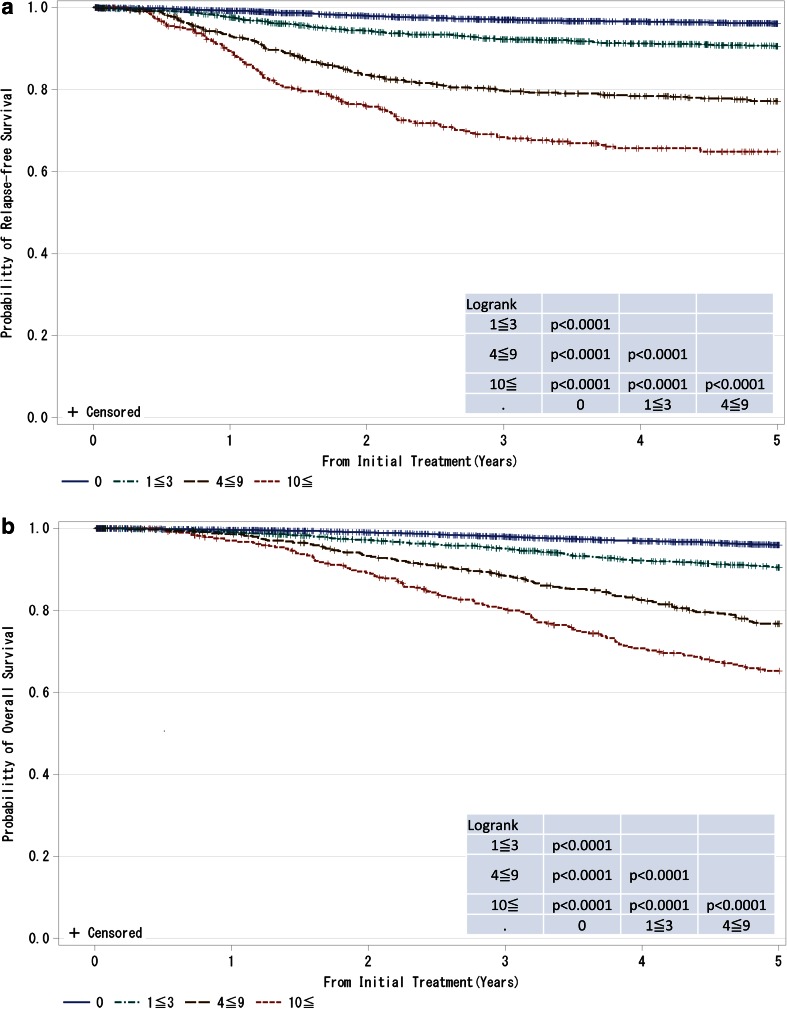
**a**, **b** Kaplan–Meier curves for relapse-free and overall survival of cases without neoadjuvant therapy by the number of metastatic lymph nodes. *P* values were calculated using the log-rank test

**Fig. 6:**
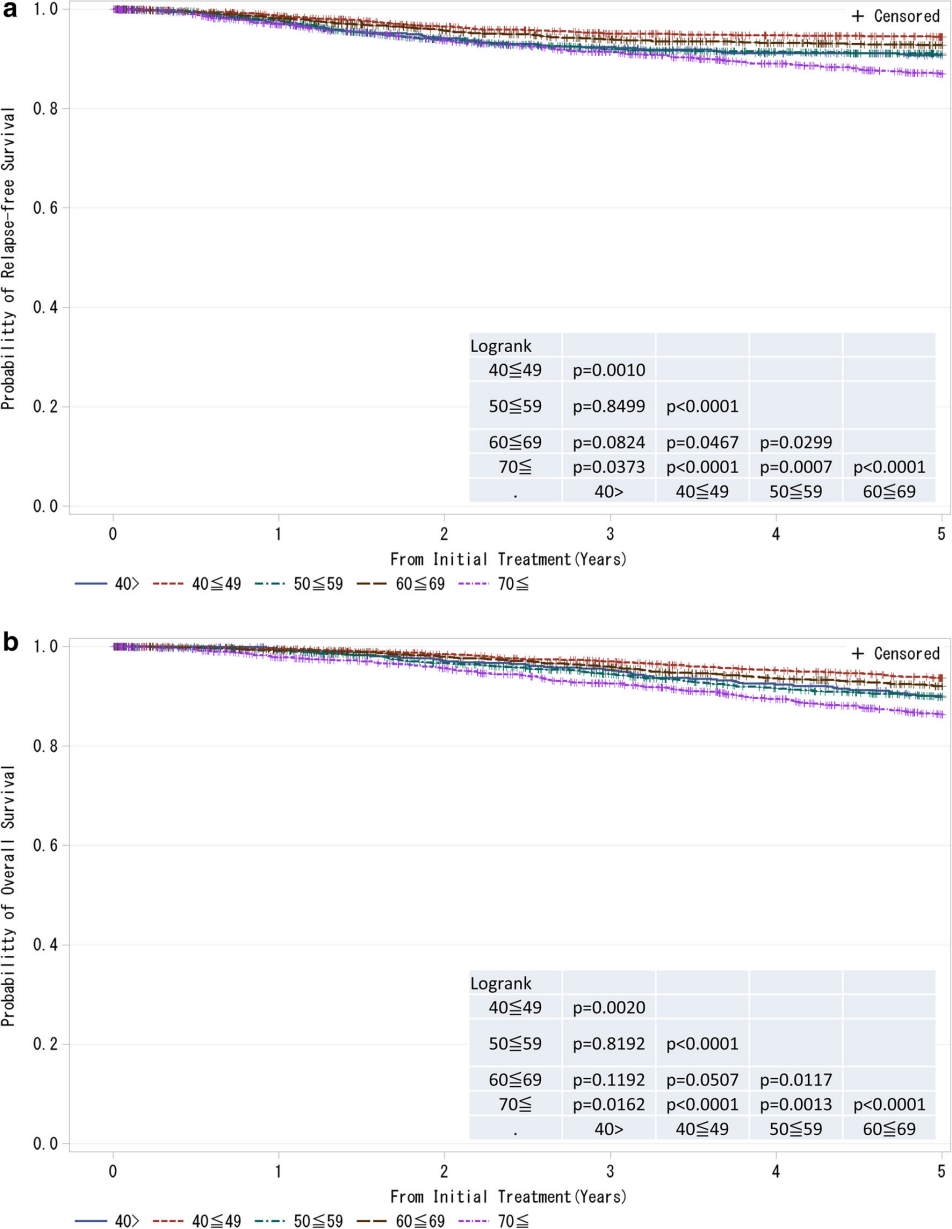
**a**, **b** Kaplan–Meier curves for relapse-free and overall survival of all cases by age. *P* values were calculated using the log-rank test

**Fig. 7:**
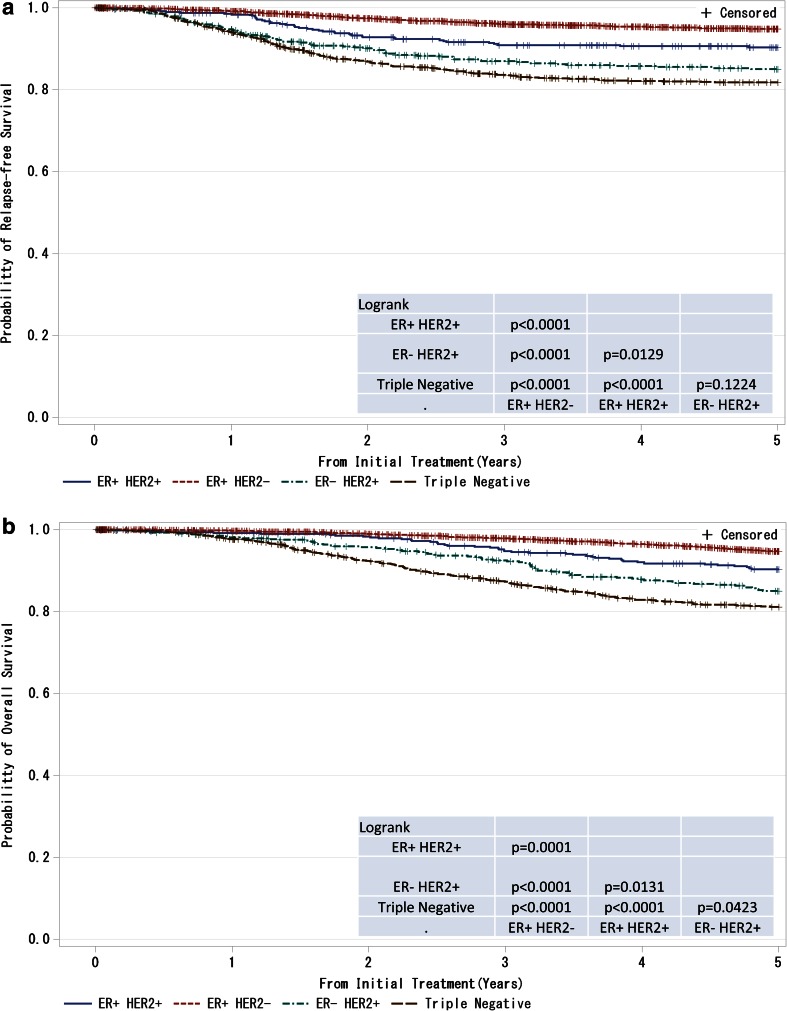
**a**, **b** Kaplan–Meier curves for relapse-free and overall survival of T1–T4, any N and M0 cases with respect to estrogen receptor (ER) status and HER2 (human epidermal growth factor receptor 2) amplification status. *P* values were calculated using the log-rank test. Relapse-free survival and overall survival of patients with respect to combined ER and HER2 status

**Fig. 8:**
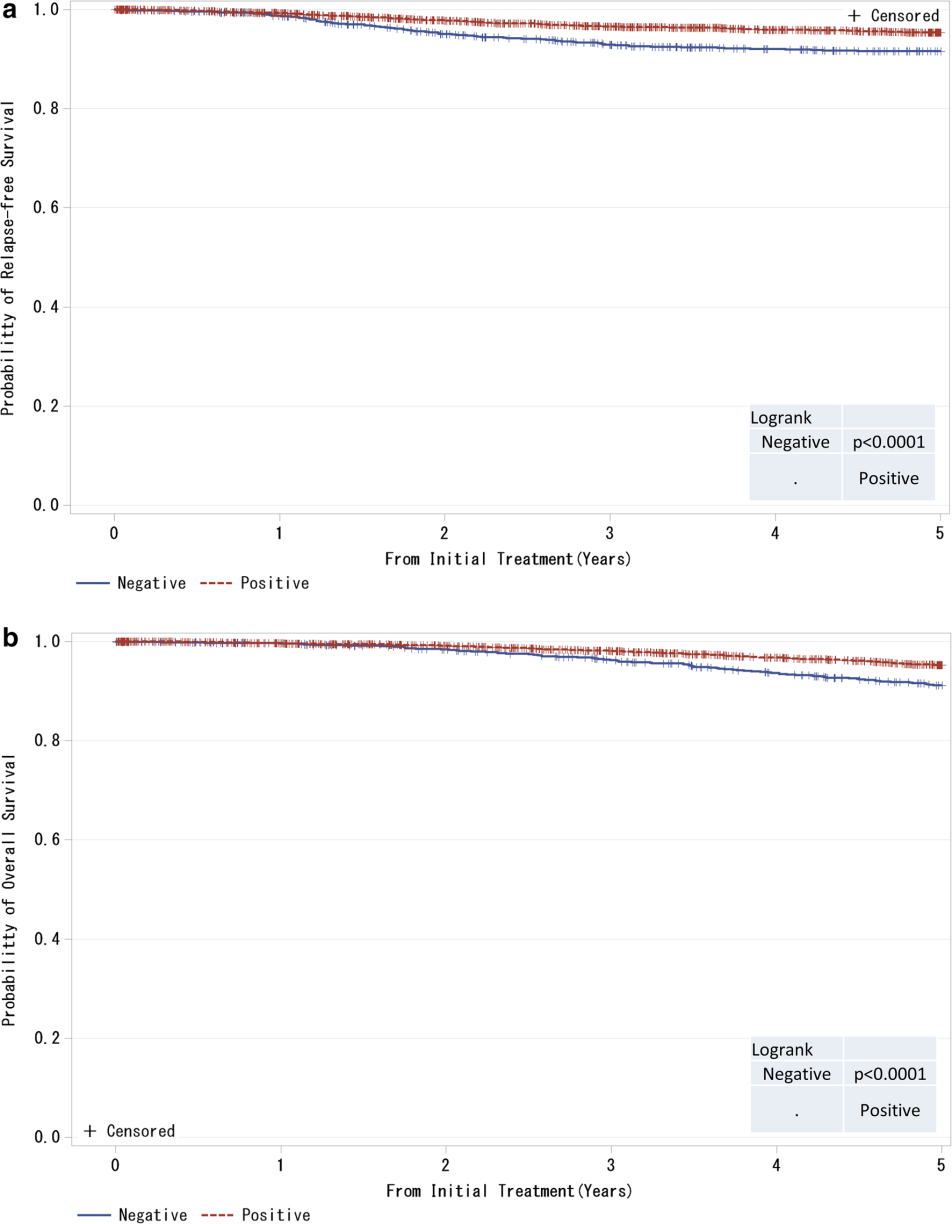
**a**, **b** Kaplan–Meier curves for relapse-free and overall survival of ER-positive and M0 cases by progesterone receptor (PgR) status. *P* values were calculated using the log-rank test

**Fig. 9:**
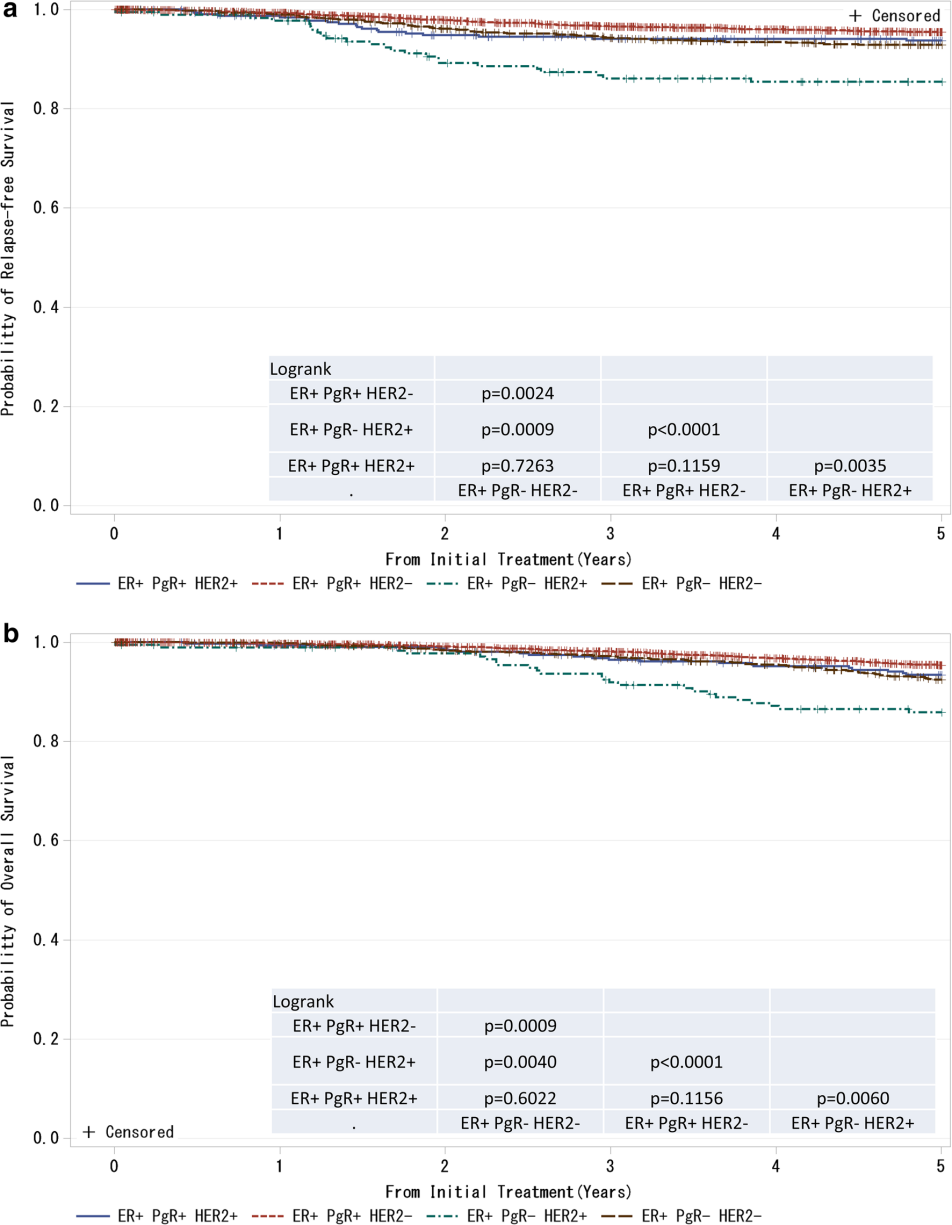
**a**, **b** Kaplan–Meier curves for relapse-free and overall survival of ER-positive and M0 cases with respect to PgR and HER2 amplifications. *P* values were calculated using the log-rank test

A total of 16,087 patients were originally registered from 317 institutions in 2004. Of these, 8585 (53.4 %) patients from 156 institutions were included in this prognostic study; we need to promote further participation in prognostic studies in the future. We believe that continuing the study based on this registration system to assess the prognosis of breast cancer will contribute to improvements in breast cancer treatment in Japan and consequently the welfare of breast cancer patients, the ultimate goal of breast cancer care.

Background characteristics of the patients are summarized in Table 1. The 5-year Disease Free Survival (DFS) was 91.5 %, and the 5-year overall survival (OS) was 90.7 % at a median follow-up of 60.0 months (range 0.0–60.0). TNM and histopathological classifications were determined using the UICC staging and WHO classification systems, respectively. TNM classification was performed according to the sixth edition of the American Joint Committee on Cancer (AJCC) staging system [1]. Histological classification was based on the WHO classification system [2]. The present report includes age- and subtype-based analyses in addition to the traditional TNM classification-based analyses. In the clinical setting, Estrogen receptor (ER), Progesterone receptor (PgR), and HER2 status, which are strong prognostic factors, have become frequently used to determine the therapeutic strategy. This trend has important implications for breast cancer treatment; therefore, we propose that subtype-based classification should be considered when the UICC’s TNM classification system is next revised. Note that during the study period, trastuzumab was rarely used because it was not covered by the Japanese National Health Insurance program as adjuvant therapy for HER2-positive breast cancer. Recently, trastuzumab has been more frequently used to treat recurrent breast cancer. Thus, we believe that data in the registry such as the relapse-free survival of patients with HER2-positive breast cancer can be an important resource for comparison with data obtained after the drug has been covered by the insurance.

**Table 1:** Patient characteristics

Age	56.99 (Mean)	12.89 (S.D.)
Tumor size(cm)	2.72 (Mean)	2.08 (S.D.)
Tumor size		
T0	139	1.62
Tis	592	6.9
T1a	52	0.61
T1b	655	7.63
T1c	2462	28.68
T2	3249	37.85
T3	396	4.61
T4	520	6.06
Unknown	520	6.06
N		
N0	6538	76.16
N1	1585	18.46
N2	277	3.23
N3	72	0.84
Unknown	113	1.32
M		
M0	8154	94.98
M1	224	2.61
Unknown	207	2.41
Stage		
0	551	6.42
I	2792	32.52
II	3466	40.37
III	725	8.44
IV	224	2.61
Unknown	827	9.63
ER		
Positive	6014	70.05
Negative	2141	24.94
Unknown	430	5.01
PgR		
Positive	4887	56.92
Negative	3227	37.59
Unknown	471	5.49
HER2		
Positive	1213	14.13
Negative	5879	68.48
Unknown	1493	17.39

The TNM classification was identified by the UICC staging system; *ER* estrogen receptor, *PgR* progesterone receptor, *HER2* human epidermal growth factor receptor 2

On January 1, 2012, the Breast Cancer Registry was incorporated in the current National Clinical Database (NCD) registration system. We appreciate the considerable support that we have received and would like to ask for continuing understanding and support of the registry.

## Electronic supplementary material

Supplementary material 1 (DOCX 31 kb)
