# Supplementary material for: Comprehensive prognostic report of the Japanese Breast Cancer Society Registry in 2004
Source: Breast Cancer. 2015 Oct 7;23:39–49. doi: 10.1007/s12282-015-0644-5 (PMC4691586; doi:10.1007/s12282-015-0644-5)
Supplement: Supplementary file 1 — Supplementary material 1 (DOCX 31 kb) [file 12282_2015_644_MOESM1_ESM.docx]

| Supplementary Table 1 Survival rate of all cases by tumor classification (cT-category) | | | | | | |
| --- | --- | --- | --- | --- | --- | --- |
| a. Relapse-free survival rate (%) | | | | | | |
| T | Number of Patients | 1 Year | 2 Year | 3 Year | 4 Year | 5 Year |
| Tis | 586 | 99.3 | 98.93 | 98.75 | 98.17 | 97.56 |
| T1a | 51 | 100 | 100 | 98 | 98 | 96 |
| T1b | 654 | 99.21 | 98.89 | 98.07 | 97.38 | 96.86 |
| T1c | 2,454 | 99.29 | 97.88 | 96.72 | 96.16 | 95.82 |
| T2 | 3,220 | 97.73 | 93.8 | 91.46 | 90.37 | 89.61 |
| T3 | 380 | 92.66 | 86.58 | 82.6 | 80.99 | 80.3 |
| T4 | 467 | 89.24 | 79.8 | 75.27 | 72.79 | 70.89 |
|  |  |  |  |  |  |  |
| b. Overall survival rate (%) | | | | | | |
| T | Number of Patients | 1 Year | 2 Year | 3 Year | 4 Year | 5 Year |
| Tis | 590 | 99.13 | 99.13 | 98.77 | 98.01 | 97.42 |
| T1a | 52 | 100 | 100 | 98 | 98 | 96 |
| T1b | 655 | 99.69 | 99.37 | 98.72 | 98.22 | 96.85 |
| T1c | 2,461 | 99.59 | 98.7 | 97.88 | 96.57 | 95.74 |
| T2 | 3,245 | 98.94 | 97.07 | 94.88 | 91.74 | 89.38 |
| T3 | 394 | 97.65 | 91.97 | 86.17 | 82.48 | 78.96 |
| T4 | 519 | 96.43 | 88.54 | 80.23 | 71.27 | 66.12 |

| Supplementary Table 2 Survival rate of all cases by regional lymph nodes status (cN-category) | | | | | | |
| --- | --- | --- | --- | --- | --- | --- |
| a. Relapse-free survival rate (%) | | | | | | |
| N | Number of Patients | 1 Year | 2 Year | 3 Year | 4 Year | 5 Year |
| N0 | 6,494 | 98.99 | 97.35 | 96.19 | 95.56 | 94.93 |
| N1 | 1,544 | 95.15 | 89.32 | 85.99 | 84.18 | 83.42 |
| N2 | 250 | 87.52 | 70.96 | 62.97 | 61.41 | 59.58 |
| N3 | 61 | 80.85 | 66.04 | 52.76 | 48.54 | 48.54 |
|  |  |  |  |  |  |  |
| b. Overall survival rate (%) | | | | | | |
| N | Number of Patients | 1 Year | 2 Year | 3 Year | 4 Year | 5 Year |
| N0 | 6,533 | 99.44 | 98.59 | 97.36 | 95.83 | 94.66 |
| N1 | 1,581 | 98.45 | 94.48 | 91.1 | 85.88 | 82.49 |
| N2 | 277 | 95.58 | 86.25 | 73.72 | 67.42 | 56.4 |
| N3 | 72 | 91.45 | 81.06 | 67.52 | 49.11 | 44.5 |

| Supplementary Table 3 Survival rate of all cases by clinical stage (UICC) | | | | | | |
| --- | --- | --- | --- | --- | --- | --- |
| a. Relapse-free survival rate (%) | | | | | | |
| Clinical stage | Number of Patients | 1 Year | 2 Year | 3 Year | 4 Year | 5 Year |
| 0 | 547 | 99.24 | 98.85 | 98.66 | 98.25 | 97.6 |
| Ⅰ | 2,785 | 99.3 | 98.4 | 97.47 | 97.06 | 96.64 |
| Ⅱ | 3,452 | 98.29 | 94.84 | 92.79 | 91.65 | 90.97 |
| Ⅲ | 708 | 91.09 | 82.07 | 76.37 | 74.77 | 73.58 |
|  |  |  |  |  |  |  |
| b. Overall survival rate (%) | | | | | | |
| Clinical stage | Number of Patients | 1 Year | 2 Year | 3 Year | 4 Year | 5 Year |
| 0 | 550 | 99.25 | 99.25 | 98.86 | 98.25 | 97.61 |
| Ⅰ | 2,791 | 99.6 | 99 | 98.32 | 97.37 | 96.59 |
| Ⅱ | 3,462 | 99.27 | 97.64 | 95.73 | 92.85 | 90.9 |
| Ⅲ | 724 | 97.31 | 91.85 | 84.77 | 78.74 | 73.04 |
| Ⅳ | 223 | 91.99 | 77.2 | 66.16 | 52.62 | 44.19 |

| Supplementary Table 4 Survival rate of cases without neoadjuvant therapy by pathological tumor size (pT size) | | | | | | |
| --- | --- | --- | --- | --- | --- | --- |
| a. Relapse-free survival rate (%) | | | | | | |
| pT size | Number of Patients | 1 Year | 2 Year | 3 Year | 4 Year | 5 Year |
| 0-5mm | 605 | 99.33 | 98.3 | 97.59 | 96.68 | 96.5 |
| 6-10mm | 912 | 99.55 | 98.52 | 97.34 | 97.22 | 96.34 |
| 11-20mm | 2,976 | 99.14 | 97.1 | 95.96 | 95.31 | 94.9 |
| 21-30mm | 1,858 | 97.86 | 94.56 | 92.54 | 91.52 | 90.58 |
| 31-40mm | 742 | 96.13 | 91.8 | 89.52 | 87.6 | 86.58 |
| 41-50mm | 401 | 93.82 | 88.99 | 85.82 | 83.98 | 83.65 |
| 51mm- | 566 | 91.87 | 84.36 | 79.61 | 78.73 | 77.55 |
|  |  |  |  |  |  |  |
| b. Overall survival rate (%) | | | | | | |
| pT size | Number of Patients | 1 Year | 2 Year | 3 Year | 4 Year | 5 Year |
| 0-5mm | 607 | 99.66 | 99.49 | 98.62 | 97.02 | 96.48 |
| 6-10mm | 916 | 99.78 | 99.1 | 98.06 | 97.58 | 96.24 |
| 11-20mm | 2,987 | 99.59 | 98.55 | 97.3 | 95.82 | 94.79 |
| 21-30mm | 1,867 | 99.07 | 97.34 | 95.56 | 92.61 | 90.53 |
| 31-40mm | 752 | 98.51 | 95.85 | 92.82 | 89.09 | 86.02 |
| 41-50mm | 413 | 98.26 | 94.68 | 90.22 | 85.59 | 81.91 |
| 51mm- | 591 | 97.21 | 91.07 | 85.19 | 79.89 | 76.16 |

| Supplementary Table 5 Survival rate of cases without neoadjuvant therapy by the number of metastatic lymph nodes | | | | | | |
| --- | --- | --- | --- | --- | --- | --- |
| a. Relapse-free survival rate (%) | | | | | | |
| n | Number of Patients | 1 Year | 2 Year | 3 Year | 4 Year | 5 Year |
| 0 | 5,102 | 99.18 | 97.9 | 97.01 | 96.54 | 96.08 |
| 1≦3 | 1,762 | 97.63 | 94.34 | 92.27 | 91.19 | 90.61 |
| 4≦9 | 633 | 93.17 | 83.51 | 79.72 | 78.38 | 77.12 |
| 10≦ | 359 | 89.32 | 75.73 | 68.33 | 65.68 | 64.85 |
|  |  |  |  |  |  |  |
| b. Overall survival rate (%) | | | | | | |
| n | Number of Patients | 1 Year | 2 Year | 3 Year | 4 Year | 5 Year |
| 0 | 5,117 | 99.58 | 98.97 | 97.99 | 96.9 | 95.95 |
| 1≦3 | 1,772 | 99.03 | 97.21 | 95.05 | 92.13 | 90.53 |
| 4≦9 | 643 | 98.58 | 93.28 | 88.37 | 82.46 | 76.73 |
| 10≦ | 374 | 97 | 89.17 | 80.24 | 70.75 | 65.23 |

| Supplementary Table 6 Survival rate of all cases by age | | | | | | |
| --- | --- | --- | --- | --- | --- | --- |
| a. Relapse-free survival rate (%) | | | | | | |
| Age | Number of Patients | 1 Year | 2 Year | 3 Year | 4 Year | 5 Year |
| 40> | 677 | 96.97 | 94.14 | 92.31 | 91.06 | 90.66 |
| 40≦49 | 1,894 | 98.65 | 96.49 | 95.04 | 94.74 | 94.43 |
| 50≦59 | 2,420 | 97.72 | 93.86 | 91.95 | 91.38 | 90.98 |
| 60≦69 | 1,922 | 98.09 | 95.63 | 93.87 | 93.22 | 92.78 |
| 70≦ | 1,535 | 96.88 | 93.68 | 91.39 | 89.01 | 86.96 |
|  |  |  |  |  |  |  |
| b. Overall survival rate (%) | | | | | | |
| Age | Number of Patients | 1 Year | 2 Year | 3 Year | 4 Year | 5 Year |
| 40> | 689 | 99.4 | 97.12 | 95.26 | 92.38 | 90.05 |
| 40≦49 | 1,915 | 99.52 | 98.49 | 97 | 95.24 | 93.73 |
| 50≦59 | 2,463 | 99.09 | 96.68 | 94.42 | 91.57 | 89.84 |
| 60≦69 | 1,946 | 99.32 | 97.88 | 95.93 | 93.58 | 92.08 |
| 70≦ | 1,554 | 97.87 | 95.65 | 92.58 | 89.47 | 86.33 |

| Supplementary Table 7 Survival rate of T1-T4, any N and M0 cases with respect to estrogen receptor (ER) status and HER2 (human epidermal growth factor receptor 2) amplification status | | | | | | |
| --- | --- | --- | --- | --- | --- | --- |
| a. Relapse-free survival rate (%) | | | | | | |
| Breast cancer subtypes | Number of Patients | 1 Year | 2 Year | 3 Year | 4 Year | 5 Year |
| ER+ HER2- | 4132 | 99.18 | 97.4 | 96.02 | 95.35 | 94.82 |
| ER+ HER2+ | 462 | 98.46 | 92.77 | 90.86 | 90.6 | 90.33 |
| ER- HER2+ | 520 | 94.73 | 90.02 | 86.91 | 85.73 | 84.98 |
| Triple Negative | 972 | 93.99 | 86.82 | 83.57 | 82.09 | 81.7 |
|  |  |  |  |  |  |  |
| b. Overall survival rate (%) | | | | | | |
| Breast cancer subtypes | Number of Patients | 1 Year | 2 Year | 3 Year | 4 Year | 5 Year |
| ER+ HER2- | 4149 | 99.71 | 98.95 | 97.87 | 96.46 | 94.67 |
| ER+ HER2+ | 467 | 99.14 | 98.26 | 94.7 | 91.96 | 90.31 |
| ER- HER2+ | 522 | 98.07 | 95.68 | 92.43 | 87.83 | 85.02 |
| Triple Negative | 979 | 97.6 | 92.3 | 87.21 | 82.84 | 81.06 |

| Supplementary Table 8 Survival rate of ER-positive and M0 cases by progesterone receptor (PgR) status | | | | | | |
| --- | --- | --- | --- | --- | --- | --- |
| a. Relapse-free survival rate (%) | | | | | | |
| PgR | Number of Patients | 1 Year | 2 Year | 3 Year | 4 Year | 5 Year |
| Positive | 4,465 | 99.29 | 97.77 | 96.57 | 95.94 | 95.32 |
| Negative | 1,172 | 98.7 | 95.03 | 92.87 | 91.98 | 91.57 |
|  |  |  |  |  |  |  |
| b. Overall survival rate (%) | | | | | | |
| PgR | Number of Patients | 1 Year | 2 Year | 3 Year | 4 Year | 5 Year |
| Positive | 4,481 | 99.64 | 99.14 | 98.09 | 96.72 | 95.23 |
| Negative | 1,184 | 99.57 | 98.35 | 96.21 | 93.64 | 91.17 |

| Supplementary Table 9 Survival rate of ER-positive and M0 cases with respect to PgR and HER2 amplifications | | | | | | |
| --- | --- | --- | --- | --- | --- | --- |
| a. Relapse-free survival rate (%) | | | | | | |
| Breast cancer subtypes | Number of Patients | 1 Year | 2 Year | 3 Year | 4 Year | 5 Year |
| ER+ PgR- HER2- | 832 | 99.02 | 96.14 | 94.3 | 93.46 | 92.88 |
| ER+ PgR+ HER2- | 3,545 | 99.33 | 97.83 | 96.62 | 95.96 | 95.47 |
| ER+ PgR- HER2+ | 178 | 97.71 | 89.23 | 86.06 | 85.38 | 85.38 |
| ER+ PgR+ HER2+ | 319 | 98.72 | 94.79 | 94.1 | 94.1 | 93.7 |
| b. Overall survival rate (%) | | | | | | |
| Breast cancer subtypes | Number of Patients | 1 Year | 2 Year | 3 Year | 4 Year | 5 Year |
| ER+ PgR- HER2- | 842 | 99.76 | 98.41 | 97.16 | 95.48 | 92.4 |
| ER+ PgR+ HER2- | 3,554 | 99.71 | 99.15 | 98.14 | 96.75 | 95.38 |
| ER+ PgR- HER2+ | 179 | 98.88 | 97.71 | 91.89 | 87.11 | 85.89 |
| ER+ PgR+ HER2+ | 323 | 99.37 | 98.73 | 96.45 | 95.11 | 93.39 |
